# Supplementary material for: Taxon-Specific Shifts in Bacterial and Archaeal Transcription of Dissolved Organic Matter Cycling Genes in a Stratified Fjord
Source: mSystems. 2021 Dec 14;6(6):e00575-21. doi: 10.1128/mSystems.00575-21 (PMC8670421; doi:10.1128/mSystems.00575-21)
Supplement: TABLE S1 [file msystems.00575-21-st001.pdf]

**Table S1** | Metatranscriptome and CTD parameter data summaries for the two vertical depth profiles in July and September 2017 at station 4 in the Gullmar fjord.

| Month | July | | | | | | | | | | September | | | | | | | | | |
|---|---|---|---|---|---|---|---|---|---|---|---|---|---|---|---|---|---|---|---|---|
| Depth [m] | 5 | | 15 | | 50 | | 75 | | 100 | | 2 | | 25 | | 55 | | 75 | | 100 | |
| Biological replicate | A | B | A | B | A | B | A | B | A | B | A | B | A | B | A | B | A | B | A | B |
| *CTD parameters* | | | | | | | | | | | | | | | | | | | | |
| Salinity | 27.22 | | 31.37 | | 33.77 | | 34.61 | | 34.68 | | 22.31 | | 33.12 | | 34.05 | | 34.55 | | 34.62 | |
| Temperature [°C] | 17.67 | | 13.6 | | 7.14 | | 7.61 | | 7.74 | | 15.97 | | 15.88 | | 8.27 | | 7.56 | | 7.63 | |
| Fluorescence | 0.54 | | 3.11 | | 0.1 | | 0.11 | | 0.15 | | 2.93 | | 0.23 | | 0.12 | | 0.13 | | 0.18 | |
| Oxygen | 5.26 | | 4.85 | | 4.13 | | 2.71 | | 2.04 | | 4.62 | | 3.92 | | 2.82 | | 2.04 | | 1.3 | |
| *Raw reads* | | | | | | | | | | | | | | | | | | | | |
| Metatranscriptomic ($\times 10^7$) | 1.39 | 1.2 | 1.82 | 1.74 | 1.72 | 1.76 | 1.42 | 2.06 | 1.55 | 1.79 | 1.58 | 1.73 | 1.89 | 2.15 | 1.95 | 1.87 | 1.62 | 1.62 | 1.96 | 1.58 |
| *Reads after QC* | | | | | | | | | | | | | | | | | | | | |
| Metatranscriptomic ($\times 10^7$) | 1.34 | 1.15 | 1.75 | 1.66 | 1.62 | 1.68 | 1.34 | 1.94 | 1.48 | 1.7 | 1.51 | 1.64 | 1.79 | 2.04 | 1.83 | 1.76 | 1.54 | 1.52 | 1.86 | 1.5 |
| % remaning | 96.1 | 95.5 | 95.7 | 95.6 | 94.3 | 95.4 | 94.7 | 94.2 | 95.1 | 94.6 | 95.7 | 95.2 | 94.6 | 95.1 | 94.1 | 94.5 | 95.1 | 93.8 | 94.6 | 94.6 |
| *Protein encoding reads* | | | | | | | | | | | | | | | | | | | | |
| Metatranscriptomic mapped to ORFs ($\times 10^6$) | 3.09 | 3.01 | 5.24 | 4.8 | 6.8 | 6.65 | 5.8 | 9.3 | 6.51 | 8.69 | 3.75 | 4.28 | 6.11 | 7.16 | 6.76 | 6.77 | 6.77 | 6.81 | 7.75 | 8.08 |
| % mapped | 23.1 | 26.2 | 30 | 28.9 | 41.9 | 39.7 | 43.2 | 48 | 44.1 | 51.2 | 24.8 | 26 | 34.1 | 35 | 36.9 | 38.4 | 44 | 44.9 | 41.8 | 54 |
| *Ribosomal RNA reads* | | | | | | | | | | | | | | | | | | | | |
| Metatranscriptomic mapped to rRNA ($\times 10^6$) | 2.01 | 2.07 | 3.28 | 2.91 | 2.55 | 3.19 | 2.74 | 3.35 | 2.8 | 1.61 | 2.54 | 2.83 | 3.15 | 3.75 | 3.37 | 3.02 | 2.62 | 2.74 | 3.51 | 1.87 |
| % rRNA | 64.9 | 68.9 | 62.5 | 60.7 | 37.5 | 47.9 | 47.2 | 36 | 43.1 | 18.5 | 67.8 | 66.2 | 51.5 | 52.4 | 49.9 | 44.6 | 38.7 | 40.3 | 45.3 | 23.2 |
| *Protein encoding reads after rRNA removal* | | | | | | | | | | | | | | | | | | | | |
| remaining after rRNA removal ($\times 10^6$) | 1.08 | 0.94 | 1.96 | 1.89 | 4.25 | 3.46 | 3.07 | 5.96 | 3.7 | 7.08 | 1.21 | 1.45 | 2.96 | 3.41 | 3.39 | 3.75 | 4.15 | 4.07 | 4.24 | 6.21 |
| % remaining after rRNA removal | 35.1 | 31.1 | 37.5 | 39.3 | 62.5 | 52.1 | 52.8 | 64 | 56.9 | 81.5 | 32.2 | 33.8 | 48.5 | 47.6 | 50.1 | 55.4 | 61.3 | 59.7 | 54.7 | 76.8 |

Abbreviations: QC, quality control; CTD parameter represent single measurements at a given depth.
